# Predictive model based on multiple immunofluorescence quantitative analysis for pathological complete response to neoadjuvant immunochemotherapy in lung squamous cell carcinoma

**DOI:** 10.3389/fonc.2024.1396439

**Published:** 2024-06-03

**Authors:** Meng Xiao, Lili Tu, Ting Zhou, Ye He, Xiaohui Li, Qiunan Zuo

**Affiliations:** The Geriatric Respiratory Department, Sichuan Provincial People’s Hospital, University of Electronic Science and Technology of China, Chengdu, China

**Keywords:** lung squamous cell carcinoma, neoadjuvant immunochemotherapy, pathological complete response, predictive model, multiple immunofluorescence quantitative analysis

## Abstract

**Objective:**

This study aims to establish a prediction model for neoadjuvant immunochemotherapy (NICT) in lung squamous cell carcinoma to guide clinical treatment.

**Methods:**

This retrospective study included 50 patients diagnosed with lung squamous cell carcinoma who received NICT. The patients were divided into the pathological complete response (PCR) group and the non-PCR group. HE staining and multiple immunofluorescence (mIF) techniques were utilized to analyze the differences in the immune microenvironment between these groups. LASSO regression and optimal subset regression were employed to identify the most significant variables and construct a prediction model.

**Results:**

The PCR group showed higher densities of lymphocyte nuclei and karyorrhexis based on HE staining. Furthermore, based on mIF analysis, the PCR group showed higher cell densities of CD8+, PD-L1+, and CD8+PD-L1+ in the tumor region, while showing lower cell densities of CD3+Foxp3+, Foxp3+, and CD163+. Logistic univariate analysis revealed CD8+PD-L1+, PD-L1+, CD8+, CD4+LAG-3+, lymphocyte nuclei, and karyorrhexis as significant factors influencing PCR. By using diverse screening methods, the three most relevant variables (CD8+, PD-L1+, and CD8+PD-L1+ in the tumor region) were selected to establish the prediction model. The model exhibited excellent performance in both the training set (AUC=0.965) and the validation set (AUC=0.786). In the validation set, In comparison to the conventional TPS scoring criteria, the model attained superior accuracy (0.85), specificity(0.67), and sensitivity (0.92).

**Conclusion:**

NICT treatment might induce anti-tumor effects by enriching immune cells and reactivating exhausted T cells. CD8+, PD-L1+, and CD8+PD-L1+ cell abundances within the tumor region have been closely associated with therapeutic efficacy. Incorporating these three variables into a predictive model allows accurate forecasting of treatment outcomes and provides a reliable basis for selecting NICT treatment strategies.

## 1 Introduction

Lung cancer is one of the most common and deadliest cancers worldwide (1). Surgical resection is the main strategy for the treatment of non-small cell lung cancer (NSCLC). However, even with complete tumor removal, NSCLC patients have poor postoperative prognosis, with a 5-year survival rate of approximately 50% for stage II and 20% for stage III (2). This unfavorable outcome may be attributed to residual tumor cells, tumor micro-metastases, or circulating tumor cells (CTCs) and circulating tumor DNA (ctDNA) causing tumor metastasis or recurrence. Even neoadjuvant chemotherapy only improves the 5-year survival rate by 5%, which is relatively limited (3, 4). Squamous cell carcinoma, compared to other subtypes of NSCLC, lacks effective therapeutic targets and has a worse prognosis. In recent years, our understanding of the role of the immune system in regulating tumor development has significantly increased, leading to a revolution in the field of cancer treatment with the emergence of neoadjuvant immunochemotherapy (NICT). It can effectively activate immune responses and potentially eliminate residual lesions or small metastatic foci (5). NICT in NSCLC has shown significant advantages in terms of short-term outcomes, such as safety, tolerability, and major pathological response (6, 7). However, not all patients benefit from NICT, which also imposes a substantial economic burden. Therefore, identifying the potential beneficiaries of NICT, excluding low responders, reducing healthcare costs, and avoiding overtreatment are urgent issues that need to be addressed.

Pathological complete response (PCR) is a critical indicator for evaluating NICT efficacy in lung squamous cell carcinoma (8).. Previous studies have aimed to identify biomarkers associated with PCR. In the context of esophageal, breast, and colorectal solid tumors, biomarkers such as PD-L1 score, tumor mutation burden (TMB), tumor-infiltrating lymphocytes (TILs), and microsatellite instability (MSI) have been considered closely linked to the effectiveness of NICT. However, the findings from certain clinical trials exhibit inconsistency and even contradictory results (9–11). These discrepancies highlight significant variations in NICT response across different tumor types and individuals, possibly attributable to the intricate nature of the tumor immune microenvironment. Therefore, analyzing the immune microenvironment could serve as a reliable approach for predicting the efficacy of NICT in lung squamous cell carcinoma.

The efficacy of anti-tumor therapy is closely related to the complex tumor immune microenvironment (TME). The complexity of the TME is determined by factors including the quantity, spatial distribution, and function of immune cells. Previous studies have shown that the distance from immune cells to tumor nests is a critical factor affecting prognosis Moreover, the separation among CD20+ B cells, CD4+ T cells, and CD8+ T cells leads to distinguished spatial immune architectures affecting the functional state of immune cells (12). Furthermore, the density of functionally suppressed CD8+ T cells can accurately predict NICT’s treatment response. One possible mechanism is to reverse the immune suppression state of exhausted killer T cells (CD8+PD-L1+ T cells) by using PD-L1 inhibitors, thereby activating immune cell killing functions and achieving pathological remission (13). Hence, analyzing the quantity, functionality, and spatial distribution of immune cells is a viable option for predicting the attainment of PCR in NICT.

With the development of multiplex immunofluorescence technology (mIF), it becomes possible to directly observe the quantity, spatial distribution, and phenotype of immune cells in the microenvironment. By leveraging machine learning, the aforementioned image features can be quantitatively analyzed. This study aims to utilize these approaches to identify and characterize immune cell features within the tumor microenvironment (TME), select the most informative features, and establish a concise and efficient predictive model for assessing the efficacy of neoadjuvant therapy. The findings will serve as a valuable reference for clinical decision-making.

## 2 Methods

### 2.1 Sample treatment

Fifty fiberoptic bronchoscopy biopsy samples of lung squamous cell carcinoma were selected for this study. The specimens were fixed in 10% neutral formalin, embedded in paraffin, sectioned using routine procedures, and subjected to hematoxylin and eosin (HE) staining. The HE staining process followed the operating instructions of an automated HE instrument. All patients underwent NICT prior to surgery. Chemotherapy regimens included single drugs or combinations of drugs such as albumin-bound paclitaxel, carboplatin, cisplatin, docetaxel, and oxaliplatin. All 50 patients received one of the following immune checkpoint inhibitors: Pembrolizumab (9 patients), Nivolumab (7 patients), Camrelizumab (26 patients), Atezolizumab (7 patients), or Durvalumab (1 patient). All patients received 1 to 4 cycles of treatment, with an average of (3.13 ± 0.58) cycles.

The mIF staining, which was completed at Genecast Biotechnology Co., Ltd., detected two panels comprising a total of 10 markers: CD4 (EP204), CD8 (SP16), PD-L1 (SP142), TIM3 (EPR22241), and LAG-3 (EPR20261) in panel 1; and CD3 (LN10), CD20 (L26), CD21 (EP3093), CD163 (10D6), and Foxp3 (236A/E7) in panel 2. CD3, CD20, and CD21 were also employed to mark tertiary lymphoid structures (TLS). CD20 is a marker of B cells, CD21 is a marker of dendritic cells, and CD163 is a marker of histiocytes. These cells are widely involved in antigen presentation and humoral immunity. CD3 is a marker of T cells, CD4 is a marker of helper T cells, CD8 is a marker of cytotoxic T cells, and Foxp3 is a marker of regulatory T cells. These cells are widely involved in cellular immunity. PD-L1, LAG-3 and TIM3 are immune checkpoint markers. And the above-mentioned immune cell markers can be used to mark the exhausted immune cells. Multiple images were obtained from serial sections of the same block per patient, and they were stained with DPAI and five markers in either panel 1 or panel 2. Each panel was detected using a 4-μm thick slide cut from FFPE NSCLC tissues. After deparaffinization and rehydration, epitope retrieval was performed by boiling the slides in Tris-EDTA buffer at 97°C for 20 min. Subsequently, endogenous peroxidase was blocked by incubation for 10 min in Antibody Block/Diluent, followed by blocking of protein in 0.05% Tween solution at 26°C for 30 min. The five antigens in each panel were then labeled by cyclic staining, which included incubation with primary and secondary antibodies, tyramine signal amplification (TSA) visualization, and removal of the TSA-antibody complex in Tris-EDTA buffer using microwave treatment at 97°C for 20 min. In each cycle, antibody labeling was performed after epitope retrieval and protein blocking as mentioned above. Following cyclic staining, each slide was counterstained with DAPI for 5 min and mounted in Pro-Long Diamond Antifade Mountant (Thermo Fisher).

### 2.2 Quantitative image analysis

The HD-Staining deep learning model, developed based on the Mask-RCNN architecture (https://github.com/matterport/Mask_RCNN), utilized 12,000 cells from pathological image patches (500 × 500 pixels) as the training set. It was validated and tested with 1,127 and 1,086 cells, respectively, confirming the reliability of the HD-Staining algorithm model (14). The HD-Staining deep learning model was used to classify and segment cell nuclei in HE images. The segmented cell nuclei were divided into six categories: tumor cells, stromal cells, lymphocytes, macrophages, red blood cells, and nuclear bleeding. Any other structures or spaces were considered background. The model then outputted the data accordingly.

The images were acquired using the TissueFAXS panoramic tissue cell imaging quantitative analysis system (TissueFAXS SL Plus S, Austria, TissueGnostics). The specific operational method was as follows: A preview of the entire slide at 2.5X magnification was conducted to determine the tissue’s position on the slide. Based on the targets’ expression in each dye channel, the optimal exposure time, exposure value, and other parameters were adjusted to determine the best scanning conditions. The sample scanning area was selected according to the results of the image preview. The selected area was then scanned at 20X magnification under the specified scanning conditions. Cell and tissue type identification, as well as protein expression quantification, were performed on panoramic images using the StrataQuest 7.1.129 image analysis software (Austria, TissueGnostics). Firstly, an intelligent algorithm was applied to segment all cells in the tissue area surrounding the nucleus. Additionally, tissue type recognition was achieved through a combination of manual training and machine learning methods, enabling the division of tissues into different regions such as tumor and stroma. Finally, protein expression was quantified by determining the average fluorescence threshold for each detection marker, which facilitated the calculation of the number of positively labeled cells. Positive cells were defined as those exhibiting an immunofluorescence signal greater than the threshold and displaying the appropriate expression pattern.

### 2.3 Statistical analysis

Logic regression, Wilcoxon test, Least Absolute Shrinkage and Selection Operator (LASSO) regression, and Optimal Subset Regression were employed for data analysis. Thirty samples were allocated as training sets, while twenty samples were designated as validation sets to construct and validate the model. Receiver Operating Characteristic (ROC) curves were utilized to assess the model’s performance. Data analysis was conducted using the R statistical software (The R Foundation, http://www.R-project.org) and the FreeStatistics statistical analysis platform. A significance level of P < 0.05 was employed.

## 3 Results

### 3.1 Pathological and clinical features

The mean age of all 50 patients was 56.00 [53.25, 66.75] years old, and 44 (88%) of them were male. In addition, 12 (24%) of the patients achieved PCR during clinical stage II-III. There were no statistically significant differences in the basic clinical characteristics between the PCR and Non-PCR groups (refer to **Table 1**).

**Table 1:** The clinicopathological characteristics of PCR group and Non-PCR group were compared.

Characteristic	Total (n = 50)	PCR (n = 38)	Non-PCR (n = 12)	p
**Gender, n (%)**				0.314
** Male**	44 (88.0)	32 (84.2)	12 (100)	
** Female**	6 (12.0)	6 (15.8)	0 (0)	
**Age, n (%)**				0.071
** ≤65**	36 (72.0)	30 (78.9)	6 (50)	
** >65**	14 (28.0)	8 (21.1)	6 (50)	
**Cigarette smoking history, n (%)**				0.621
** Yes**	44 (88.0)	34 (89.5)	10 (83.3)	
** No**	6 (12.0)	4 (10.5)	2 (16.7)	
**Alcohol drinking history, n (%)**				1
** Yes**	34 (68.0)	26 (68.4)	8 (66.7)	
** No**	16 (32.0)	12 (31.6)	4 (33.3)	
**Family history, n (%)**				1
** Yes**	9 (18.0)	7 (18.4)	2 (16.7)	
** No**	41 (82.0)	31 (81.6)	10 (83.3)	
**cT, n (%)**				0.226
** T2**	32 (64.0)	22 (57.9)	10 (83.3)	
** T3**	12 (24.0)	10 (26.3)	2 (16.7)	
** T4**	6 (12.0)	6 (15.8)	0 (0)	
**cN, n (%)**				0.496
** 1**	31 (62.0)	25 (65.8)	6 (50)	
** 2**	19 (38.0)	13 (34.2)	6 (50)	
**stage, n (%)**				0.485
** II**	21 (42.0)	17 (44.7)	4 (33.3)	
** III**	29 (58.0)	21 (55.3)	8 (66.7)	
**Differentiation, n (%)**				0.077
** Well**	11 (22.0)	9 (23.7)	2 (16.7)	
** Middle**	29 (58.0)	19 (50)	10 (83.3)	
** Poor**	10 (20.0)	10 (26.3)	0 (0)	
**Primary tumor location, n (%)**				0.294
** Central**	33 (66.0)	27 (71.1)	6 (50)	
** Peripheral**	17 (34.0)	11 (28.9)	6 (50)	

### 3.2 HE images quantitative analysis results

**Figure 1:**
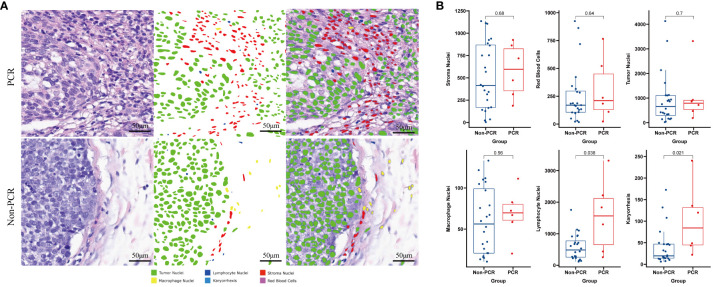
Machine learning identifies different cell types on HE-stained slices and compares the differences between PCR and Non-PCR groups **(A)**. The PCR group exhibits higher densities of Lymphocyte Nuclei and Karyorrhexis **(B)**.

We employed machine learning to utilize the results of quantitative analysis of Hematoxylin and Eosin (HE) images for specific cell type identification in HE slices. The PCR group exhibited significantly higher densities of Lymphocyte Nuclei and Karyorrhexis (P<0.05) (refer to **Figure 1B**), with a more pronounced observation of this phenomenon in the tumor interior and at the invasive margin (refer to **Figure 1A**).

### 3.3 Multiple immunofluorescence quantitative analysis results

**Figure 2:**
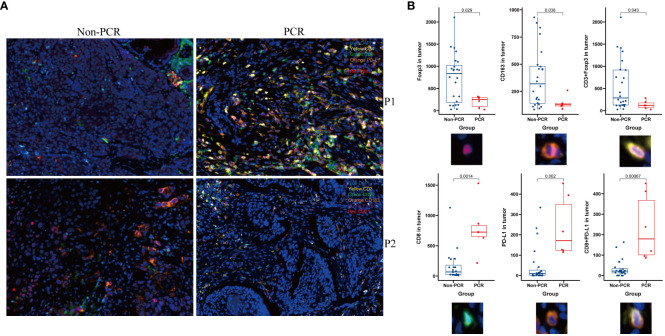
Multiple immunofluorescence technology was used to analyze the immune microenvironment. Panel 1and Panel 2 (P1, P2)Schematic diagram showed The PCR group exhibited a higher abundance of immune cell infiltration and PD-L1 expression within and around the tumor nests, along with fewer macrophages and Foxp3-positive regulatory T cells **(A)** and image quantitative analysis results demonstrated that in the tumor area, the densities of CD8, PD-L1+, and CD8+PD-L1+ cells were significantly higher in the PCR group compared to the Non-PCR group, while the densities of CD3+Foxp3+, Foxp3+, and CD163+ cells were significantly lower in the PCR group than in the Non-PCR group **(B)**.

**Figure 3:**
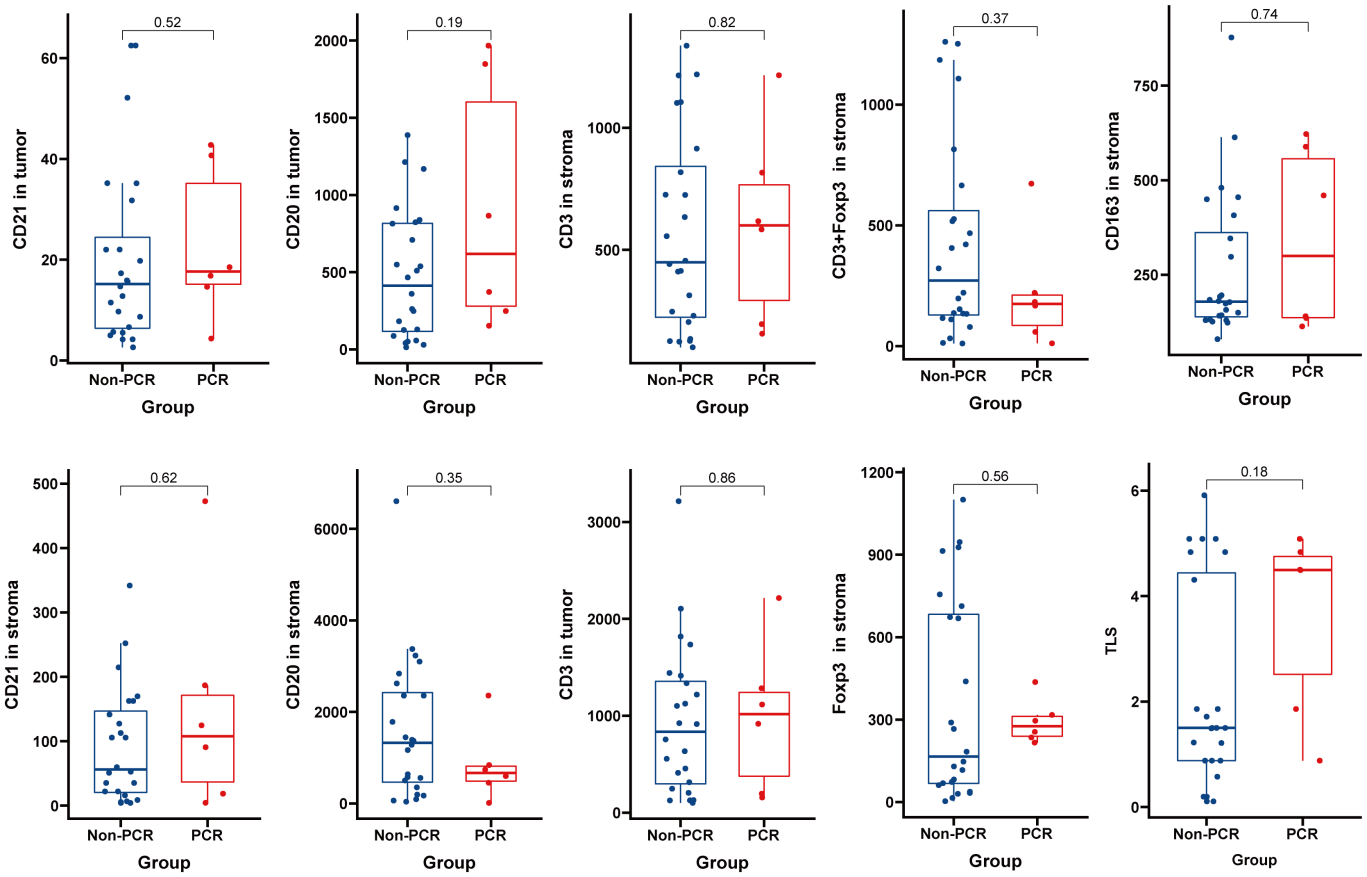
No Significant Differences Found in Multiplex Immunofluorescence Panel 1 Results.

**Figure 4:**
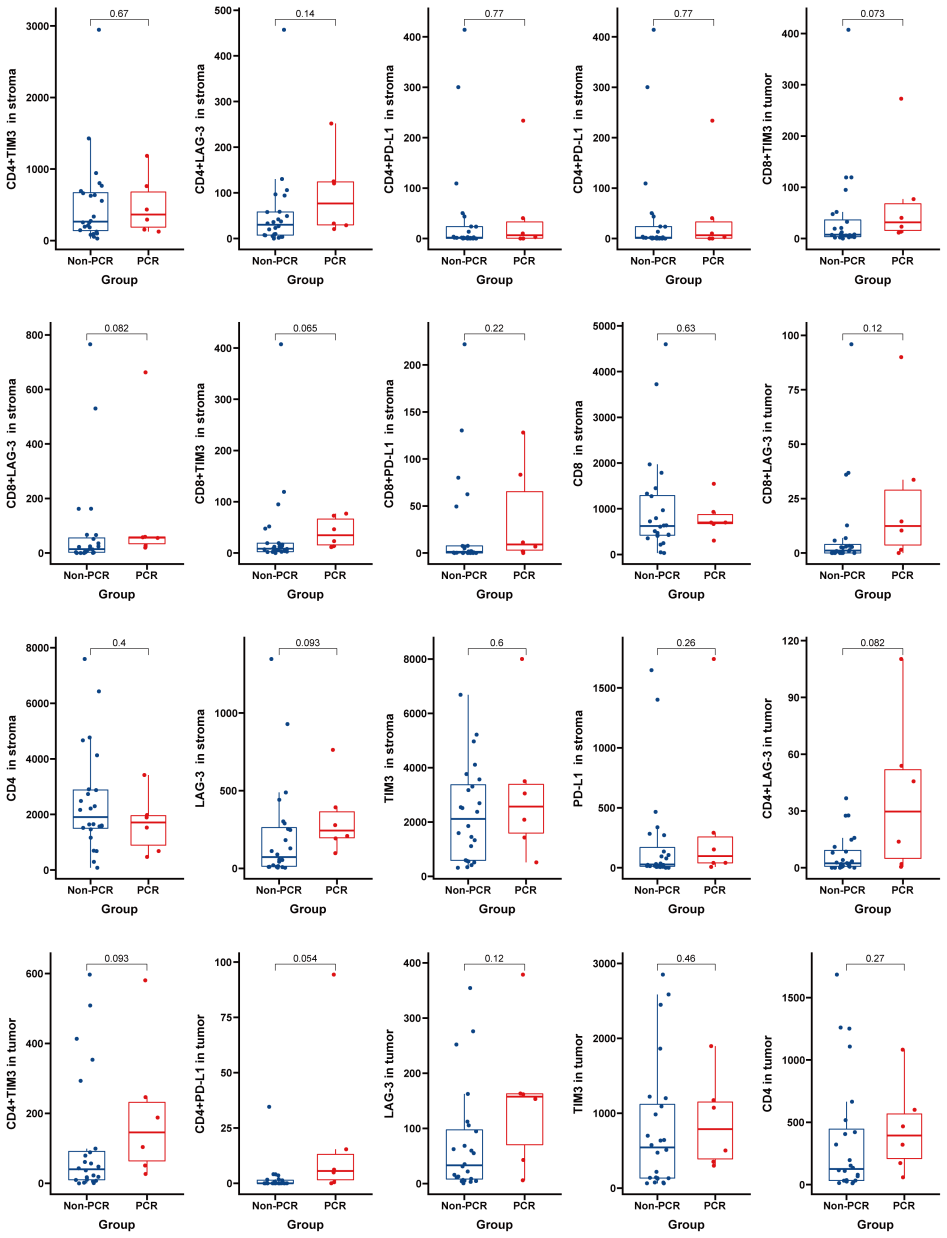
No Significant Differences Found in Multiplex Immunofluorescence Panel 2 Results.

Panel 1 (P1) detection indicators included PD-L1, LAG3, TIM3, CD4, and CD8. The detection indexes of Panel 2 (P2) included CD20, CD21, CD3, CD163, and Foxp3. Common positive areas of CD3, CD20, and CD21 indicated tertiary lymphoid structure (TLS). The PCR group in Panel 1 (top right) exhibited more abundant immune cell infiltration and a higher PD-L1 expression rate in and around the tumor nest, while the non-PCR group in Panel 1 (top left) showed more sparse immune cell infiltration and a lower PD-L1 expression rate in and around the tumor nest. The PCR group (lower right) had fewer macrophages and FOXP3-positive regulatory T cells in and around the tumor nest, whereas the non-PCR group (lower left) had more macrophages and FOXP3-positive regulatory T cells in and around the tumor nest (refer to **Figure 2**). Quantitative analysis of Panel 2 revealed that the cell density of CD8+, PD-L1+, and CD8+PD-1+ in the PCR group was significantly higher than that in the non-PCR group in the tumor area (P<0.05). There was no statistical difference in TLS density between the two groups. Quantitative analysis of Panel 1 demonstrated that CD3+Foxp3+, Foxp3+, and the cell density of CD163+ in the PCR group were significantly lower than those in the non-PCR group (P < 0.05) (refer to **Figure 2**). The cell densities of CD4+TIM3+, CD4+PD-L1+, CD8+TIM3+, and CD4+LAG-3+ in the PCR group were higher than those in the non-PCR group. In the stroma region, CD8+LAG-3+, CD8+TIM3+ and LAG-3+ cell density in the PCR group exhibited a higher trend compared to the non-PCR group (refer to **Figures 3**, **4**).

### 3.4 Univariate and multivariate analyses

Univariate and multivariate analyses revealed that CD8+PD-L1+, PD-L1+, CD8+, CD4+LAG-3+, lymphocyte nuclei and karyorrhexis in the tumor region were independent factors influencing pathological complete response (PCR) (refer to **Table 2**).

**Table 2:** Logistic regression univariate and multivariate analysis.

Variable	Univariate Analysis		Multivariate Analysis	
	OR 95CI	P value	OR 95CI	adj.P value
**Tumor region CD3+**	1 (1~1)	0.89		
**Tumor region CD20+**	1 (1~1)	0.096		
**Tumor region CD21+**	1.01 (0.96~1.06)	0.715		
**Tumor region CD163+**	0.99 (0.98~1)	0.116		
**Tumor region Foxp3+**	1 (0.99~1)	0.065		
**Tumor region CD3+Foxp3+**	0.99 (0.99~1)	0.185		
**Stroma region CD3+**	1 (1~1)	0.877		
**Stroma region CD20+**	1 (1~1)	0.254		
**Stroma region Foxp3+**	1 (1~1)	0.644		
**Stroma region CD163+**	1 (1~1.01)	0.393		
**Stroma region CD21+**	1 (1~1.01)	0.27		
**Stroma region CD3+Foxp3+**	1 (0.99~1)	0.262		
**Stroma region CD4+**	1 (1~1)	0.669		
**Stroma region CD8+**	1.01 (1~1.01)	0.01	0.97 (0~ Inf)	<0.001
**Tumor region TIM3+**	1 (1~1)	0.796		
**Tumor region LAG3+**	1.01 (1~1.01)	0.128		
**Tumor region PD-L1+**	1.01 (1~1.02)	0.011	1.24 (0~Inf)	<0.001
**Tumor region CD4+PD-L1+**	1.06 (0.97~1.16)	0.201		
**Tumor region CD8+PD-L1+**	1.03 (1~1.06)	0.023	1.61 (0~Inf)	<0.001
**Tumor region CD8+TIM3+**	1 (0.99~1.01)	0.439		
**Tumor region CD8+LAG3+**	1.02 (0.99~1.06)	0.18		
**Tumor region CD4+LAG3+**	1.07 (1~1.13)	0.038	1.26(0~Inf)	<0.001
**Tumor region CD4+TIM3+**	1 (1~1.01)	0.32		
**Stroma region CD4+**	1 (1~1)	0.32		
**Stroma region CD8+**	1 (1~1)	0.668		
**Stroma region TIM3+**	1 (1~1)	0.378		
**Stroma region LAG3+**	1 (1~1)	0.44		
**Stroma region PD-L1+**	1 (1~1)	0.44		
**Stroma region CD4+PD-L1+**	1 (0.99~1.01)	0.83		
**Stroma region CD8+PD-L1+**	1 (0.99~1.02)	0.551		
**Stroma region CD8+TIM3+**	1 (0.99~1.01)	0.895		
**Stroma region CD8+LAG-3+**	1 (1~1.01)	0.474		
**Stroma region CD4+LAG-3+**	1 (1~1.01)	0.347		
**Stroma region CD4+TIM3+**	1 (1~1)	0.926		
**Gender Male**	12155660.62 (0~Inf)	0.994		
**Age >65**	3.8 (0.58~24.88)	0.164		
**Cigarette smoking history**	1.67 (0.16~17.26)	0.668		
**Alcohol drinking history**	0.82 (0.12~5.57)	0.842		
**Family history**	1 (0.09~11.03)	0.999		
**Differentiation poor- moderately**	1.92 (0.18~20.82)	0.591		
**Primary tumor location central-type**	0.63 (0.06~7.03)	0.703		
**Lymphocyte Nuclei**	1 (1~1)	0.023	1.05 (0~Inf)	<0.001
**Karyorrhexis**	1.02 (1~1.03)	0.041	1.39 (0~Inf)	<0.001
**Stroma Nuclei**	1 (1~1)	0.697		
**Macrophage Nuclei**	1.01 (0.98~1.03)	0.608		
**Tumor Nuclei**	1 (1~1)	0.724		
**Red Blood Cells**	1 (1~1)	0.631		

“Inf” means positive infinity.

### 3.5 Establishment and verification of the prediction model

**Figure 5:**
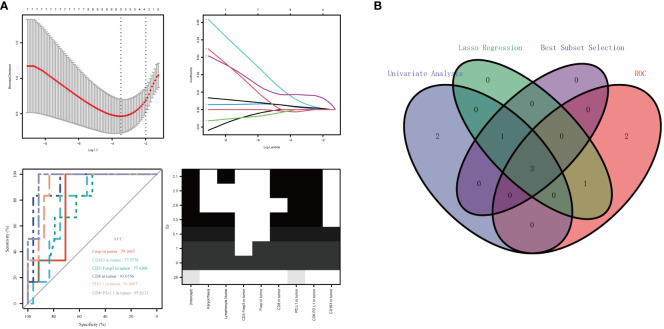
Variable selection was performed using ROC curve analysis, LASSO regression analysis, and optimal subset regression analysis **(A)**. The intersection of the selected variables was visualized in a Venn diagram, which revealed that three variables were most reliable for predicting PCR **(B)**.

**Figure 6:**
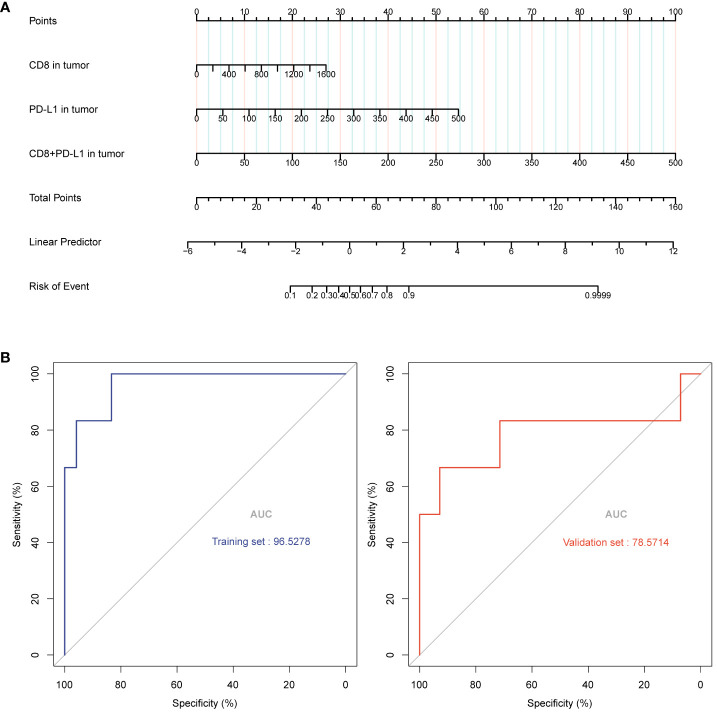
A predictive model was constructed using the selected three variables, and a line graph was obtained to predict patient PCR **(A)**. The model achieved an AUC value of 0.965 in the training set and an AUC value of 0.786 in the validation set **(B)**.

Six significantly different variables from the Non-PCR and PCR groups in panels 1 and 2 were analyzed for their predictive value in neoadjuvant therapy response using ROC curves. The AUC values, in descending order, were as follows: Tumor region CD8+PD-L1+ (95.8333%), Tumor region PD-L1 +(91.6667%), Tumor region CD8 +(93.0556%), Tumor region Foxp3+ (79.1667%), Tumor region CD163+ (77.7778%), and Tumor region CD3+Foxp3+ (77.4306%). Following LASSO regression analysis of all variables, the λ coefficient decreased with an increasing number of variables. Five variables with a non-zero coefficient were selected at the optimal value. The five variables selected were Tumor region CD8+, Tumor region PD-L1+, Tumor region CD8+PD-L1+, Tumor region Foxp3+, and Lymphocyte Nuclei, respectively. After conducting optimal subset regression analysis on all variables, when the Mallows Cp coefficient reached a minimum of 2.1, four variables were identified: Tumor region CD8+, Tumor region PD-L1+, Tumor region CD8+PD-L1+, and Lymphocyte Nuclei (refer to **Figure 5**). The common variables identified by different methods were visualized in a Venn diagram (refer to **Figure 5**). Subsequently, three variables were selected to establish a model with high clinical applicability, and a nomogram was developed for predicting PCR in patients (refer to **Figure 6**). The AUC value of the model reached 0.965 in the training set and 0.786 in the verification set (**Figure 6**). Through the hosmer-lemeshow model fitting test, it was considered that there was no significant difference between the model prediction result and the actual result (X^2^ = 11.234 P = 0.189). Compared to the traditional PD-L1 scoring standards of TPS > 1% and TPS > 50% (15), the predictive model demonstrated improved accuracy, sensitivity, and specificity (refer to **Table 3**).

**Table 3:** Comparison of predictive efficacy.

	Model	TPS 1	TPS 50
**prediction sensitivity**	0.92	1.00	0.40
**prediction specificity**	0.67	0.13	0.55
**prediction accuracy**	0.85	0.4	0.65

## 4 Discussion

We utilized multiplex immunofluorescence technology and image quantitative analysis techniques to examine the pre-treatment immune microenvironment of lung squamous cell carcinoma patients. Our analysis revealed disparities in the immune microenvironment between the PCR and non-PCR groups, specifically in terms of immune cell quantity, distribution, and function. We identified the most valuable differences for predicting the effect of NICT treatment, namely the density of CD8+ T cells, PD-L1+ T cells, and PD-L1+CD8+T cells within the tumor nest (rather than the stroma). The prediction model established using these differences performed well in both the training and validation sets, providing an accurate and easy-to-implement plan for clinical prediction of NICT efficacy.

Initially, we analyzed the spatial distribution of immune cells and identified their predominant concentration within tumor nests and invasive margins. Likewise, the differences in markers, including CD8+, PD-L1+, Foxp3+, and CD163+, were primarily observed in these regions. Importantly, no statistically significant differences were observed in the stroma. Consequently, we hypothesized that immune cells responsible for immune function were primarily distributed within the tumor rather than the stromal area. In a study investigating neoadjuvant therapy for breast cancer, researchers discovered a notable association between the quantity of immune cells within tumor nests and pathological response, whereas no such correlation was observed within the stroma (16). Similarly, a separate study focusing on gastric cancer reported a significant relationship between the quantity of immune cells infiltrating tumor nests and invasive margins and patient overall survival (OS) (17). Collectively, these findings suggest that immune cells exert their anti-tumor effects primarily within the tumor nests and invasive margins.

Additionally, we performed an analysis of immune cell densities in this study. The PCR group exhibited higher quantities of CD20+ B lymphocytes, CD4+ helper T lymphocytes, and CD8+ killer T lymphocytes, while demonstrating lower levels of Foxp3+ regulatory T cells and CD163+ M2 macrophages. These findings suggest that both humoral immunity and cellular immunity play crucial roles in anti-tumor activity when there is increased immune cell infiltration. Conversely, the presence of regulatory T cells and M2 macrophages hindered the anti-tumor effect within the immune microenvironment. Notably, previous research indicated that a 10% rise in infiltrating immune cells (TILs) in breast cancer reduced the risk of death by 20% (18). Furthermore, a study focusing on T3N0 esophageal squamous cell carcinoma reaffirmed the close correlation between the quantity of TILs and overall survival (OS) within each subgroup (19). Another study illustrated the predictive value of various TILs subpopulations in melanoma for patient response to immunotherapy (20). Moreover, regulatory T cells have the capability to suppress bodily immunity (21), consequently impeding the body’s anti-tumor activity by inhibiting the activation and proliferation of reactive T cells within the body’s microenvironment. Considering the collective findings from prior studies and our own, we have compelling grounds to suspect that the quantity of immune cells and macrophages may represent crucial factors influencing the response to neoadjuvant therapy.

Lastly, we conducted an analysis of cell functions. Additionally, our study reveals higher levels of CD20+ cell density and TLS numbers in the PCR group, although this disparity did not reach statistical significance. Notably, CD20+ B lymphocytes not only contribute to humoral immunity but also form a critical component of tertiary lymphoid structures. Numerous studies underscore the significance of CD20+ B cells and TLS in tumor immunochemotherapy (22), thereby indirectly elucidating the elevated levels of CD20+ B cells and TLS in the PCR group. However, T cells appear to constitute the primary anti-tumor component. The densities of CD8+ cells in the PCR group are significantly greater than those in the other groups, suggesting that a higher quantity of killer T cells corresponds to a more pronounced pathological response.

Our study corroborates previous reports indicating that a higher quantity of effector T cells is associated with pathological complete remission of breast cancer. This association could be attributed to the direct involvement of CD8+ T cells in tumor eradication (23). Furthermore, the density of CD8+PD-L1 depleting killer cells was higher in the PCR group in our study, indicating a greater presence of these depleting T cells exerting an anti-tumor effect through the reactivation of immune checkpoint inhibitors (23). While previous studies suggested that CD8+ killer T cells were the primary anti-tumor cells, recent research has demonstrated that CD4+ T cells are also capable of secreting granase B to eliminate cancer cells and enhancing the killing function of CD8+ T cells through the secretion of interleukin-2 (IL-2) (24). While previous studies suggested that CD8+ killer T cells were the primary anti-tumor cells, recent research has demonstrated that CD4+ T cells are also capable of secreting granase B to eliminate cancer cells and enhancing the killing function of CD8+ T cells through the secretion of interleukin-2 (IL-2) (25). These findings indicate the significant roles of both CD4+ and CD8+ T cells in the anti-tumor process, highlighting their potential for cooperative interactions (26). According to the univariate analysis in this study, CD4+LAG-3+ appeared to be a significant influencing factor for PCR, providing support for this hypothesis. However, not all T cells contribute to immune killing. Immune killing by Foxp3+ regulatory T cells is regulated by negative feedback, and an excessive presence of regulatory T cells inhibits immune killing while promoting tumor escape (27). This study found a high density of Foxp3 and CD3+Foxp3+ in the non-PCR group, providing evidence for the aforementioned perspective. The function of M2 macrophages in restricting immune response and promoting angiogenesis (28) indirectly *facilitates* immune escape and tumor metastasis. The higher presence of CD163+ macrophages in the non-PCR group in this study aligns with this perspective. In summary, disparities are observed between the PCR group and the non-PCR group in the density, spatial distribution, and function of immune cells, potentially contributing to the occurrence of PCR. Building upon the aforementioned analysis, we employ optimal subset regression and LASSO regression to identify the three most effective variables: CD8+, CD8+PD-L1+, and PD-L1+ for improved prediction of PCR. Subsequently, a PCR prediction model *is developed* and internally validated. The training set exhibits excellent performance, while there *is* a noticeable decline in performance on the validation set. This may be due to issues such as overfitting and a limited number of samples. The study is conducted in a single center, and the lack of external validation is a limitation of this research. Nevertheless, compared to traditional TPS > 1 or TPS > 50 scores, the model demonstrates strong performance in improving sensitivity, specificity, and accuracy. This provides a reliable basis for selecting clinical treatment strategies.

## Data availability statement

The raw data supporting the conclusions of this article will be made available by the authors, without undue reservation.

## Ethics statement

The studies involving humans were approved by Sichuan Provincial People’s Hospital, University of Electronic Science and Technology of China. The studies were conducted in accordance with the local legislation and institutional requirements. The ethics committee/institutional review board waived the requirement of written informed consent for participation from the participants or the participants’ legal guardians/next of kin because Utilizing medical records and biological specimens obtained from previous clinical diagnoses and treatments.

## Author contributions

MX: Conceptualization, Data curation, Formal analysis, Investigation, Methodology, Project administration, Software, Validation, Writing – original draft, Writing – review & editing. LT: Data curation, Formal analysis, Software, Writing – review & editing. TZ: Data curation, Investigation, Methodology, Writing – review & editing. YH: Data curation, Investigation, Writing – review & editing. XL: Conceptualization, Supervision, Validation, Writing – review & editing. QZ: Conceptualization, Data curation, Investigation, Methodology, Project administration, Software, Supervision, Validation, Writing – original draft, Writing – review & editing.
